# Effects of Olive Cake on the Performance, Digestibility, Blood Parameters, and Intestinal Villi of Bísaro Pigs

**DOI:** 10.3390/ani15081131

**Published:** 2025-04-14

**Authors:** Jessica Paié-Ribeiro, Divanildo Outor-Monteiro, Cristina Guedes, Maria José Gomes, José Teixeira, Alfredo Teixeira, Victor Pinheiro

**Affiliations:** 1 Animal Science Department, University of Trás-os-Montes and Alto Douro (UTAD), 5000-801 Vila Real, Portugal; divanildo@utad.pt (D.O.-M.); cguedes@utad.pt (C.G.); mjmg@utad.pt (M.J.G.); joseteixeira@utad.pt (J.T.); vpinheir@utad.pt (V.P.); 2 Veterinary and Animal Research Centre (CECAV), University of Trás-os-Montes and Alto Douro, 5000-801 Vila Real, Portugal; 3 AL4Animals, Quinta de Prados, 5000-801 Vila Real, Portugal; 4 Mountain Research Center (CIMO), Polytechnic Instituto of Bragança, Campus de Santa Apolónia, 5300-253 Bragança, Portugal; teixeira@ipb.pt

**Keywords:** olive by-product, autochthonous breed, two-phase centrifugation, circular economy

## Abstract

Olive oil production generates by-products, such as olive cake (OC), which can be used in animal feed, reducing the environmental impact. This study assessed the effects of OC in pig diets on intestinal morphometry and blood parameters. Twenty-four Bísaro pigs were randomly assigned to three treatments: OC0 (control), OC15 (15% dehydrated OC), and OC25 (25% dehydrated OC) for 93 days. The apparent total tract digestibility (ATTD) of dry matter (DM), organic matter (OM), crude protein (CP), crude fat (CF), and fiber (NDF) was evaluated through total feces collection. No significant differences in average daily gain (ADG) or feed conversion ratio (FCR) were found (*p* > 0.05). However, average daily feed intake (ADFI) increased from 2.84 kg (control) to 3.46 kg (OC15) and 3.64 kg (OC25). OC inclusion increased the ADFI of CF (90.8 g to 171.5 g and 169.2 g) and NDF (586.8 g to 896.6 g and 1115.4 g). The ATTD of all parameters decreased with 25% OC. The digestible intake of DM, OM, and CP was unaffected, but CF intake was higher in OC-fed groups (*p* = 0.005). Histomorphometric analysis revealed no significant differences in villi size. Lymphocyte counts were higher in OC25 (*p* < 0.05). This study suggests that up to 15% OC inclusion is feasible without compromising the pigs’ performance.

## Simple Summary

This study evaluated using two-phase olive cake (OC) as a potential additive in pig feed. Pigs were fed diets incorporating 0, 15, and 25% OC, and the effects on growth performance, digestibility, intestinal villi, and blood parameters were assessed. The results showed that the inclusion of OC in the diet of Bísaro pigs did not affect weight gain or the feed conversion ratio but did increase daily feed consumption. Nutrient digestibility was reduced with 25% OC, but the increase in intake compensated for this fall. Intestinal morphology was not altered, and blood parameters remained normal, except for an increase in lymphocytes in the 25% OC group. Including up to 15% OC is feasible without compromising the animals’ performance.

## 1. Introduction

The circular economy has been globally promoted as a sustainable model to close resource, material, and energy cycles. This approach is achieved through recycling, reusing products and components, and minimizing waste. This paradigm seeks to transform environmental challenges into economic opportunities in the agri-food sector, where by-product reutilization has become a key strategy [1,2].

Livestock production faces multiple sustainability constraints, including the scarcity of arable land, water, and fertile soils, as well as the impacts of climate change, biodiversity loss, and competition for resources between human food and biofuel production. The efficient use of available resources is crucial to ensure the sector’s long-term viability. One of the most promising strategies is incorporating agro-industrial by-products—particularly those that do not compete with human food—into animal feed formulations [3].

The olive industry generates substantial by-products, which, if not properly managed, can pose environmental risks. For every 1000 kg of olives processed, approximately 800 kg of waste is produced. Olive oil extraction relies on three primary methods: the traditional pressing process, the two-phase centrifugal system, and the three-phase extraction system [4,5,6].

The chemical composition of olive cake (OC) varies depending on climate conditions and extraction techniques, which influence the proportions of its components (pericarp, mesocarp, endocarp, and stone). Due to its high fiber content—rich in lignin—and moderate protein levels (around 10%), much of which is bound to fiber, OC generally has low nutritional value for most animal species [7].

Despite this, olive by-products have demonstrated potential as alternative feed ingredients, particularly for pigs in the growth and finishing phases [8], ruminants [9], rabbits [10], and poultry [11]. In the case of the Bísaro pig—an indigenous breed traditionally raised in semi-extensive systems—feeding practices primarily rely on locally available resources, reducing the carbon footprint associated with long-distance feed transport [12]. Their diet commonly includes food industry by-products alongside agricultural crops such as cereals, tubers, vegetables, and fruits. In regions with significant olive oil production, incorporating OC into pig diets has emerged as a sustainable strategy, promoting environmental conservation and the circular economy [13].

Recent results from our previous research confirmed the feasibility of incorporating olive cake (OC) into the diets of growing Bísaro pigs, revealing that moderate inclusion levels—using various types of OC over extended feeding periods—did not impair growth performance or nutrient digestibility [14]. These findings support the continued investigation of OC as a sustainable feed ingredient. Therefore, the present study builds upon this foundation to evaluate the effects of including two-phase OC at two dietary levels—15% (OC15) and 25% (OC25)—compared to a control diet (OC0). The aim is to assess the impact on productive performance, nutrient digestibility, intestinal villus morphometry, and blood parameters. Although olive oil by-products, such as OC, have been explored in various animal species, their specific application in pig nutrition—particularly in the Bísaro breed—remains underexplored. By advancing this research, we contribute to the sustainable valorization of agro-industrial residues and promote environmentally responsible livestock production systems.

## 2. Materials and Methods

This study was conducted at the Experimental Swine Unit of the University of Trás-os-Montes and Alto Douro (UTAD BioLab Sus) in Vila Real, Portugal. The handling of the animals adhered to Portuguese animal welfare legislation for experimental research (Decree-Law No. 1/2019, 10 January) and Directive 2010/63/EU of the European Parliament and the Council of 22 September 2010. The study protocol (2253-e-DZ-2022) was approved by the Animal Welfare Body (ORBEA) of UTAD.

### 2.1. Animals and Diets

The diets were formulated in a commercial feed unit using a hammer mill and horizontal mixer and were supplied in meal form. In the experimental trial, twenty-four Bísaro pigs (12 castrated males and 12 females, 1-year-old, with an average live weight of 91.6 ± 3.3 kg in the fattening phase) were housed in pairs and randomly assigned to three treatments, differing in the level of olive cake (OC) inclusion from the two-phase extraction system (two-phase OC): OC0—control diet; OC15—control diet plus 15% OC; and OC25—control diet plus 25% OC. Each treatment group comprised 8 animals (4 castrated males and 4 females), ensuring a balanced gender distribution and similar body weight across the groups. To achieve this, animals were first stratified by gender and body weight and then randomly assigned to the treatments. This method aimed to minimize variability related to sex and body weight.

Animals were housed in pens of two, providing 8 replicates per treatment for weight data and 4 replicates per treatment for feed intake measurements. The first six days of the experiment were dedicated to the animals’ adaptation to the housing conditions and experimental diets. The entire trial lasted 93 days.

### 2.2. Bromatological Analysis

The diet samples and each OC used in this study were dried in a forced-air oven at 50 °C. After drying, they were ground using a 1 mm sieve to ensure homogenization and prepare them for chemical analysis. The contents of dry matter (DM), organic matter (OM), and crude fat (CF) in the OC were evaluated according to the methods established by the AOAC (Association of Official Analytical Chemists) [15]. Crude protein (CP) content was determined by multiplying nitrogen content using a conversion factor of 6.25. The analysis of neutral detergent fiber (NDF), acid detergent fiber (ADF), and acid detergent lignin (ADL) fractions was conducted using the procedures outlined by Robertson and Van Soest [16].

### 2.3. Fatty Acid Analysis

The fatty acid profile of the experimental diet samples was analyzed using gas chromatography, following the Folch method. The fatty acid methyl esters (FAMEs) were examined using an Agilent Technologies Spain GC-Agilent 6890N gas chromatograph (Madrid, Spain) equipped with a flame ionization detector and an HP 7683 automatic sample injector. The fatty acid profile was expressed as the proportion of saturated, monounsaturated, and polyunsaturated fatty acids (SFAs, MUFAs, and PUFAs, respectively) in grams per 100 g of fatty acids (%). The analysis was conducted using the ISO 12966-2:2022 standard [17].

### 2.4. Phosphorus and Phytic Acid Content

The phytic acid content in the samples was measured using the procedure described in the Megazyme commercial kit (K-PHYT, Bray, Ireland) [18]. The quantification process involved acid extraction of the samples, followed by enzymatic treatment with phytase and phosphatase to release phosphorus. Each sample was analyzed in triplicate, and the total phosphate released was determined through a colorimetric reaction with ammonium molybdate. The formation of molybdenum blue in this reaction is proportional to the inorganic phosphate (Pi) present, with quantification based on the increase in absorbance at 655 nm. Phosphorus levels were calculated using a calibration curve generated from standards with known phosphorus concentrations. The results were expressed as grams of phosphorus per 100 g of sample (g P/100 g).

### 2.5. Granulometry of Diets

To determine the particle size of all the diets fed to the animals, we followed the protocol described by Paié-Ribeiro et al. [14]. The particle size distribution of the diets was important for assessing their physical properties and understanding how this parameter can be used to optimize the feeding of the animals. Subsamples were taken from all the diets used in this experiment until a sample of approximately 1 kg was formed. Subsequently, representative samples of all the diets were taken, labeled, and properly stored. Each sample was individually subjected to particle size analysis, in duplicate, using a Retsch analytical sieve shaker, model AS 200 basic (Haan, Germany), with four sieves of different sizes (2.3 mm, 1.18 mm, 0.6 mm, and 0.3 mm). The material retained on each sieve was carefully collected and weighed, and the percentages of particles retained on each sieve were recorded to obtain the size distribution curve for each sample.

### 2.6. Digestibility Trial

The total fecal collection method is the gold standard for estimating pig diets’ energy values and nutrient digestibility [19]. The feces were collected in full in three daily periods over three consecutive days, stored in labeled plastic bags, and frozen until analysis. For preparation, the samples were dried in an oven at 50 °C until the dry matter (DM) content stabilized. After this stabilization, a representative sample of material from the three collection days was prepared for each animal. These samples were ground in a mill with a 1 mm sieve for chemical analyses and to determine the digestibility coefficient of the different fractions of the diet (DM, OM, NDF, CP, and CF).

### 2.7. Collection and Analysis of Blood Samples

According to standard procedures described by Rosa et al. [20], blood samples were collected following an overnight fast of at least 12 h. Approximately 10 mL of venous blood was obtained from the jugular vein using sterile vacutainer tubes, with the volume adjusted according to the number and type of analyses planned. For biochemical analysis, samples were collected in tubes without anticoagulants to obtain serum. These samples were immediately centrifuged using the JP Selecta Centromix BLT centrifuge (Barcelona, Espanha) for 5 min at 2618× *g*, and the serum was aliquoted into 2 mL tubes, properly labeled, and stored at −20 °C until analysis. Biochemical parameters—including total protein, aspartate aminotransferase (AST), alanine aminotransferase (ALT), gamma-glutamyl transferase (GGT), alkaline phosphatase (ALP), total bilirubin, and direct bilirubin—were determined using the DiaSys Respons^®^920 Clinical Chemistry Analyzer (Holzheim, Germany), following the manufacturer’s protocols. The leftover plasma was then utilized for protein electrophoresis, performed on the Elephor8S automated analyzer, following the manufacturer’s instructions.

In parallel, blood samples for hematological analysis were collected in tubes containing heparin as an anticoagulant. Hematological parameters were analyzed using the ProCyte Dx^®^ (IDEXX) hematology analyzer (Westbrook, ME, USA), which provided a complete blood count (CBC), including red and white blood cell indices, platelet parameters, and differential leukocyte counts. Thus, a single blood sampling session per animal was sufficient to perform the hematological, biochemical, and serological assessments required for this study.

### 2.8. Measurements of Intestinal Villi

To carry out a detailed analysis of the intestinal mucosa, tissue samples from the caecum and ileum were collected at the time of slaughter, according to the protocol described by Teixeira et al. [21]. Each segment measured approximately 10 cm long and was immediately placed in vials containing a 10% neutral formalin solution, ensuring adequate fixation for subsequent processing. After fixation, the samples were submitted to an automated tissue processor for dehydration, diaphanization, and impregnation, and then embedded in paraffin blocks. The paraffin blocks containing the tissues were cut on a microtome, obtaining sections between 1 and 10 μm thick. The sections were stained using the standard hematoxylin and eosin (HE) protocol, allowing clear visualization of the tissue structures under the microscope. Morphometric parameters such as the villi’s height, width, and depth were measured to analyze the intestinal mucosa.

**Figure 1:**
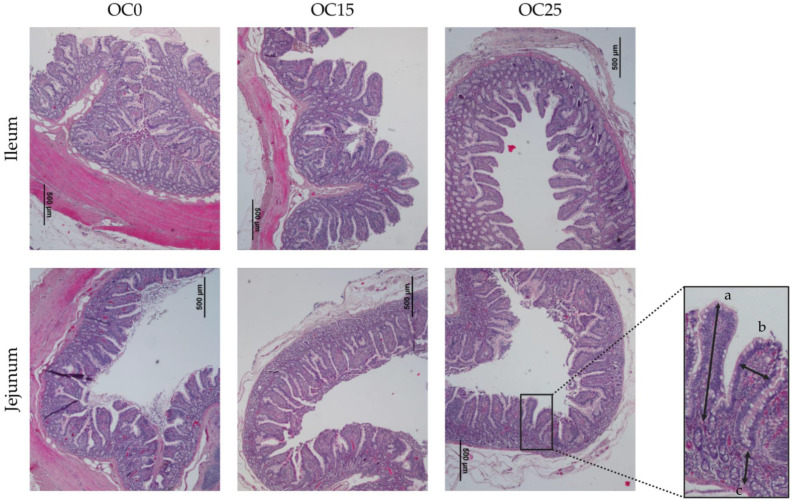
Six representative images of histomorphometric analyses of intestinal villi in two segments of the small intestine (jejunum and ileum) from animals subjected to the three experimental diets: OC0, OC15, and OC25. (a) Villi height (µm); (b) villi width (µm); (c) crypt depth (µm).

These measurements were taken using the ImageJ program (version 1.53a, National Institutes of Health, Bethesda, MD, USA), a widely used software for analyzing bio-medical images. The software made it possible to precisely identify the contours of the villi and obtain exact measurements, providing reliable quantitative data. Three images were captured for each sample (Figure 1), and five villi were measured in each image, totaling fifteen measurements per sample.

Based on these measurements, the intestinal absorption area was calculated, providing detailed information on the functionality and structural integrity of the intestinal tissue. The measurements taken included the height of the villi (VH), the width of the villi at two specific points (in the upper region (top of the villi) and the lower region (near the crypt)), and the depth of the crypts (CD). Based on this data, the absorption area of the villi (aa) was calculated using the equation below, where r represents the radius and l is the length of the villi.

$$a\;a=\frac{4\;\pi r^{2}}{2}+2\;\pi rl\;$$

### 2.9. Statistical Analysis

Data were analyzed using the statistical package JMP^®^ Pro 17.1.0 by 2023 SAS Institute Inc. © (Cary, NC, USA). Each pen (housing two animals) was considered the experimental unit for feed intake measurements, while individual animals were treated as the experimental unit for live weight, blood parameter, digestibility, and villi measurements. The data obtained were checked for normality using the Shapiro–Wilk test and subjected to variance analysis (ANOVA) with diet as a factor and a multiple-range test (Tukey’s test) for a *p*-value < 0.05.

An additional one-way ANOVA with polynomial contrasts was performed using IBM SPSS Statistics Version 30.0.0.0 (Chicago, IL, USA, 2024) to evaluate the linear and quadratic effects of the three dietary treatments.

All results are presented in tables as mean values ± the standard error of the mean (SEM). Superscripts indicate significant differences between treatments; non-significant differences are omitted for clarity. Tables also include *p*-values for all tested effects.

## 3. Results

### 3.1. Chemical Composition of Olive Cakes and Diets

Paié-Ribeiro et al. described the chemical characterization of OC earlier [22,23]. The chemical composition, total phosphorus, and phytic acid content of the different diets used are shown in Table 1. The levels of NDF and ADF increased considerably with the addition of OC. NDF went from 19.4% at OC0 to 27.8 at OC15 and 33.5 at OC25. ADF went from 6.9 to 14.0 and 18.8, respectively. The ADL content followed the same trend, being 1.25% for OC0 and 4.94 and 7.41 for OC15 and OC25, respectively. In addition, CF increased from 5.6 in OC0 to 6.1 in OC15 and 6.4 in OC25. On the other hand, the CP content decreased as the inclusion of OC increased. The values were 14.9% for the control treatment, 13.6 for OC15, and 12.7 for OC25.

Total phosphorus values decreased as the percentage of OC increased, with 0.359 g/100 g for OC0, 0.337 for OC15, and 0.240 for OC25. Similarly, the phytic acid content decreased, showing values of 1.27 g/100 g, 1.20, and 0.85 for OC0, OC15, and OC25, respectively.

### 3.2. Fatty Acid Profile

Table 2 shows the fatty acid profile of the three experimental diets (OC0, OC15, and OC25), with the composition expressed as a percentage of total fatty acids. The composition varied according to the level of inclusion of OC. There was a progressive reduction in the content of SFA, from 18.4 g/100 g in the OC0 to 18.1 g/100 g in the OC15 diet and 16.3 g/100 g in the OC25 diet.

MUFAs increased significantly with the inclusion of OC, mainly due to the increase in oleic acid (C18:1n-9), whose content went from 24.1 g/100 g in OC0 to 26.4 g/100 g in OC15 and 38.9 g/100 g in OC25. Consequently, the ΣMUFA values rose from 26.6 g/100 g in the control diet to 28.9 g/100 g and 41.71 g/100 g in the diets with 15% and 25% OC, respectively.

On the other hand, the fraction of PUFAs decreased with the inclusion of OC, from 33.2 g/100 g in OC0 to 32.0 g/100 g in OC15 and 25.11 g/100 g in OC25. This reduction was mainly influenced by the lower concentration of linoleic acid (C18:2n-6), which varied from 31.0 g/100 g in OC0 to 29.8 g/100 g in OC15 and 23.3 g/100 g in OC25.

The PUFA/SFA ratio also decreased as the level of OC inclusion increased, falling from 1.80 in the OC0 diet to 1.76 and 1.54 in the diets with 15% and 25% OC, respectively. In addition, the n-6/n-3 ratio followed the same trend, going from 16.0 to 15.7 and 14.1 in the different treatments.

### 3.3. Granulometry Analysis of Olive Cakes and Diets

Analyzing the particle size of OCs and the experimental diets revealed significant differences in granulometry (Table 3). As expected, the geometric mean diameter of the particles was substantially greater in the OC analyzed alone (645.7 µm) compared to the experimental diets. Among the diets, a progressive increase in the geometric mean diameter was observed as the level of OC increased, ranging from 205.1 µm in the OC0 diet to 250.4 µm in the OC15 diet and reaching 294.4 µm in the OC25 diet.

The fineness modulus also increased with the inclusion of OC, from 0.98 mm in the control diet to 1.27 mm and 1.50 mm in the diets with 15% and 25% OC, respectively.

Evaluation of the uniformity index showed that, in OCs, the highest proportion of particles was in the >2 mm range (31.8%), followed by particles between 0.60 and 2 mm (50.5%) and particles < 0.60 mm (17.7%). In the experimental diets, the fraction of particles > 2 mm was non-existent in the control diet, but increased with the inclusion of OC, totaling 7.04% in the OC15 diet and 9.30% in OC25. The intermediate fraction (0.60 to 2 mm) remained relatively constant between the diets (42.4% to 43.7%), while the fraction of smaller particles (<0.60 mm) decreased with the addition of OC, from 57.6% in the control diet to 49.2% in the diets with 15% and 25% OC.

These results indicate that including OC in the diet increases the average particle size and fineness modulus, alters the particle size distribution, reduces the fraction of fine particles, and increases the presence of larger particles.

### 3.4. Performance Measurements

The results of the study on the performance of Bísaro pigs fed different levels of OC are shown in Table 4. The pigs’ initial live weight (iLW) showed no significant differences between the treatments, with average values of 91.4 kg for OC0, 92.1 kg for OC15, and 91.4 kg for OC25. Final live weight (fLW) also showed no significant differences in the analysis of variance (ANOVA). Still, a significant quadratic effect was observed (*p* = 0.018), indicating that the relationship between OC level and fLW is not linear, suggesting that including 15% OC was more favorable for fLW.

The average daily gain (ADG) showed no significant differences between the treatments, with values of 0.526 g for OC0, 0.629 g for OC15, and 0.515 g for OC25. However, the average daily feed intake (ADFI) showed significant differences between treatments (*p* = 0.036), with pigs fed OC15 and OC25 diets consuming significantly more feed (3.46 g and 3.64 g, respectively) compared to OC0 (2.84 g). In addition, a significant linear effect (*p* = 0.013) was observed on ADFI, indicating that feed consumption increased linearly with increasing levels of OC in the diet. The feed conversion ratio (FCR) showed no significant differences between treatments, with values of 4.64 for OC0, 5.01 for OC15, and 6.59 for OC25.

In summary, the incorporation of OC into the diet of Bísaro pigs mainly affected daily feed consumption, with a significant increase in the OC15 and OC25 treatments and no effects on fLW, ADG, and FCR.

### 3.5. Digestibility Results

The results of intake, apparent total tract digestibility (ATTD), and digestible intake in Bísaro pigs fed different levels of OC are shown in Table 5. Regarding intake, there were no significant differences in DM, OM, and CP between treatments. However, the intake of CF and NDF showed significant differences (*p* = 0.002 for CF and *p* = 0.002 for NDF), with higher values in the OC15 and OC25 treatments compared to OC0. CF intake was 90.8 g/day for OC0, 171.5 g/day for OC15, and 169.2 g/day for OC25. NDF intake was 586.8 g/day for OC0, 896.6 g/day for OC15, and 1115.4 g/day for OC25. A significant linear effect (*p* = 0.002) and a quadratic effect (*p* = 0.025) were observed for CF intake. For NDF intake, a significant linear effect was observed (*p* = 0.001).

Significant differences were observed in the ATTD for all the components evaluated. DM digestibility was 83.7% for OC0, 78.2% for OC15, and 71.9% for OC25 (*p* = 0.003), with a significant linear effect (*p* = 0.001). OM digestibility was 85.8% for OC0, 79.5% for OC15, and 72.8% for OC25 (*p* = 0.002), with a significant linear effect (*p* = <0.001). CP digestibility was 85.5% for OC0, 84.5% for OC15, and 79.3% for OC25 (*p* = 0.030), with a significant linear effect (*p* = 0.014). CF digestibility was 91.2% for OC0, 89.5% for OC15, and 82.6% for OC25 (*p* = 0.002), with a significant linear effect (*p* = 0.001). NDF digestibility was 61.4% for OC0, 46.9% for OC15, and 37.4% for OC25 (*p* = 0.016), with a significant linear effect (*p* = 0.005). ANOVA was utilized for all the parameter differences between the OC0 and OC25 treatments.

Regarding digestible intake, no significant differences were observed for DM, OM, CP, and NDF. However, the digestible CF intake showed significant differences (*p* = 0.005) between OC0 and the other two treatments, with values of 82.5 g/day for OC0, 153.8 g/day for OC15, and 140.0 g/day for OC25. A significant linear effect (*p* = 0.007) and a quadratic effect (*p* = 0.016) were observed for digestible CF intake.

### 3.6. Hematological Parameters

The blood profile of the Bísaro pigs fed different levels of OC (Table 6) showed that most of the parameters evaluated did not change significantly (*p* > 0.05). Although some statistical differences were observed (*p* < 0.05), all the values remained within the reference standards.

Blood lipids, total cholesterol, HDL, LDL, and triglyceride levels showed no significant variations between the treatments. However, an upward trend (*p* = 0.055) concerning HDL gradually increased as OC was incorporated. For erythrocytes, ANOVA showed differences between treatments (*p* = 0.023) and OC0 had lower values than OC25, with a linear effect (*p* = 0.007). No significant differences were detected for parameters such as hematocrit, hemoglobin, MCV, MCH, MCHC, RDW, and reticulocytes.

The total leucocyte count did not differ between treatments (*p* > 0.05). However, the percentage of lymphocytes was significantly higher in the OC15 and OC25 groups compared to the OC0 group (*p* = 0.010), and the lymphocyte count was also higher in these groups (*p* = 0.016). The percentage of neutrophils and eosinophils is lower as the level of the OC increases. The lymphocytes showed differences between treatments (*p* = 0.011) with increased values for higher OC levels and a linear effect (*p* = 0.004). The monocyte showed differences between treatments (*p* = 0.033), with higher values for OC0 and lower values for OC15 and a quadratic effect (*p* = 0.015). The eosinophils showed linear effects (*p* = 0.034) with lower values when the percentage of OC increases. The total count of lymphocytes was significantly affected by diet (*p* = 0.006), with significant differences between diet OC0 and the other two treatments, with higher values and linear (*p* = 0.013) and quadratic (*p* = 0.025) effects.

Concerning platelets, the count was significantly lower in the OC15 group compared to the OC0 group (*p* = 0.024) with a linear effect (*p* = 0.025). Plateletcrit (%) follows the same trend with lower values for the OC15 group compared to OC0 (*p* = 0.020), with linear (*p* = 0.042) and quadratic (*p* = 0.038) effects. As for coagulation parameters such as prothrombin time (PT), INR, and APTT, no significant differences were observed. Liver enzymes, including ALT, GGT, AST, and alkaline phosphatase, were not significantly affected by diet, nor were total bilirubin levels.

### 3.7. Analyses of the Intestinal Mucosa: Height and Width of Villi and Depth of Crypt

Table 7 presents the results of the intestinal villi measurements. There were no significant differences (*p* > 0.05) between treatments for all measurements in the ileum and jejunum.

## 4. Discussion

Olive cake (OC), a by-product of olive oil extraction, has gained attention for its potential use in animal feed and industrial applications. Its chemical composition varies significantly depending on residual oil content, processing methods, and the proportion of skin, pulp, and water [24,25]. However, its inclusion in animal diets has been limited due to the presence of anti-nutritional compounds (e.g., phytic acid, polyphenols, and tannins), which can reduce fiber digestibility, impair feed efficiency, and affect palatability [26,27,28,29]. In addition, the animals’ acceptance of this ingredient can be a limiting factor due to compounds that affect its palatability [26].

Incorporating OC into pig diets increases fiber content, particularly crude fiber (CF) and neutral detergent fiber (NDF), as observed in previous studies [30,31]. This by-product has a high fiber and fat concentration, as previously reported by Paié-Ribeiro et al. [22]. Our study observed reduced total phosphorus and phytic acid as OC was incorporated. Previous research shows low levels of total phosphorous [22,32] and phytic acid [33].

The inclusion of OC in pig diets has been investigated due to its potential benefits for sustainability and meat quality [34]. OC is rich in beneficial compounds and can impact pork products’ fatty acid composition and oxidative stability [33]. Adding OC to pig diets increased MUFAs, mainly due to increased oleic acid (C18:1 n9). These results are beneficial because MUFAs, such as oleic acid, have antioxidant properties and are beneficial for human cardiovascular health. The pig industry has invested in genetics, management, and nutrition to reduce fat accumulation in pigs while seeking to adjust the lipid composition of pig products to meet human nutritional demands. The lipid profile of the diet strongly influences the composition of fatty acids in pig adipose tissue. In the case of autochthonous breeds, known for their meat quality characteristics and high levels of intramuscular fat, modifying the diet can be an effective strategy for improving the lipid quality of pork products while preserving their traditional sensory properties [35,36].

Particle size is another critical factor. The ideal size of feed particles varies between 500 and 1600 µm, depending on the production stage and the diet’s composition. Particles smaller than 400 µm can cause damage to the gastric mucosa, increasing the risk of ulcers and keratinization, which compromises the animals’ health. On the other hand, larger particles can decrease the digestibility and palatability of the feed [37,38].

The inclusion of OC in pig diets can influence their food preferences due to the textural properties and palatability of the food. By nature, pigs tend to prefer less complex and more crumbly foods. Thus, the addition of OC can hinder feed acceptance, as it alters the texture of the feed, making it more fibrous [39]. In our study, the feed was given in meal form to optimize the incorporation of OC since, when it was included like a topping in a pelleted diet, the animals tended to select only the feed, leaving the OC aside.

The results obtained for the mean diameter were 205.1 (OC0), 250.4 (OC15), and 294.4 (OC25), which reflects the effect of the OC (645.7). The same trend is visible for the fineness modulus. The OC has a high percentage of large particles (<2 mm), and this effect is reflected in the diet parameters. As shown by Calleb et al. [40], pigs prefer larger particles, and they observed that animals prefer corn with particles between 500 and 700 µm over finer ones [37]. Additional studies by De Jong et al. [41] and Nemechek et al. [42] confirm this trend, indicating that reducing the particle size to less than 400 μm decreases feed consumption. In addition, decreasing particle size generally reduces the feed intake index in finishing pigs, especially when feed is offered in meal form, as mentioned by Kippert et al. [43].

By-products from the olive oil industry have been widely studied for inclusion in diets for growing and finishing pigs [30,44]. They stand out for their nutritional composition, such as their high CF content, unsaturated fatty acids, and bioactive compounds known for their antioxidant properties, as previously reported by Paié-Ribeiro et al. [23]. Despite these, their high lignin content, associated with a low crude protein concentration, has been a limiting factor for inclusion in diet formulations. However, some studies have tested levels of up to 10% and have shown satisfactory results [30,45,46]. Liotta et al. [44] observed that the inclusion of 5% OC in the diet of Pietrain pigs improved weight gain and the FCR compared to the control diet. Joven et al. [45] also reported better growth rates and feed consumption in pigs fed 5% or 10% OC, while inclusion of 15% resulted in poorer performance. This result partially corroborates our study observations. Still, we worked with a breed more tolerant of OCs. Only the inclusion level of 25% generated a drop in performance, as evidenced by the quadratic effect on fLW (*p* = 0.018), the linear effect on ADFI (*p* = 0.013), and the increase in the FCR.

Diets with a higher fiber content generally have lower energy digestibility, which can result in an increase in feed intake to compensate for the lower energy availability. However, this is not always enough to sustain growth [47,48]. In our results, we observed a significant increase in daily feed intake (ADFI), with values of 2.84 g (OC0), 3.46 g (OC15), and 3.64 g (OC25), with significant effects (*p* < 0.05). These results suggest that as OC inclusion increases, there is a compensation in feed intake to cope with the lower energy digestibility. In addition, the effects of particle sizes that were previously discussed can contribute to this higher feed intake. However, the growth of the animals did not show a proportional correspondence since the inclusion of 25% OC reduced fLW.

In our results, the FCR was 4.64 (OC0), 5.01 (OC15), and 6.59 (OC25), showing that as the inclusion of OC increased, the FCR also increased, which can be attributed to the lower efficiency of nutrient utilization with higher levels of OC inclusion. These values are in line with those of Santos and Silva et al. [49] (5.45) and Martins et al. [50] (5.2) for Bísara pigs. Although the FCR values are high, it is essential to consider that the Bísaro breed has specific genetic characteristics that can result in a lower efficiency in the utilization of nutrients from the feed, which is common in indigenous breeds at this age and weight. These animals have slower growth, high maintenance requirements, and significant fat accumulation. The meat and products derived from these animals are traditionally recognized for their excellent quality and suitability for processing, which justifies their economic value.

Thus, the performance observed in the OC15 group reinforces the hypothesis that OC can be a well-tolerated source of fiber, favoring adequate growth rates and feed consumption. Our results align with reports in the literature, which indicate that including OCs of up to 15% in pig diets does not negatively affect growth performance, average daily gain, or feed conversion ratio in local Bísaro-breed animals [30].

In this study, digestibility was calculated using the total feces collection method. Incorporating different levels of OC into the diet of Bísaro pigs significantly affects the digestibility of DM, OM, CP, CF, and NDF, with reductions observed as the level of OC in the diet increases. In addition, the intake of CF and NDF was significantly higher in the OC15 and OC25 treatments. The linear and quadratic effects indicate that these changes follow specific patterns in response to the increased level of OC in the diet.

The total feces collection method assumes that, after a prolonged adaptation period, the pigs achieve a constant feed intake and feces production during the collection period [19,51]. However, several studies have shown that dietary fiber content and diet type can influence the rate of gastrointestinal emptying and the rate of digesta passage [19,52,53,54,55,56].

Dietary fiber, commonly defined as the indigestible part of the diet, has long been recognized as a nutritionally relevant and beneficial component for health [57,58]. However, a predominant concern for monogastric animals such as pigs is that diets high in fiber are associated with reduced nutrient utilization and lower net energy values since dietary fiber cannot be broken down by endogenous digestive enzymes [59,60].

The increasing use of fiber-rich ingredients in pig diets is often seen as a strategy for reducing feed costs in pig production [48], as well as improving body metabolism and intestinal health [61,62,63], promoting animal welfare [5], and reducing ammonia emissions from manure [64]. However, despite these benefits, including fiber-rich ingredients in diets can also hurt the ATTD [19,65,66,67]. Reduced digestibility results in diets with lower energy concentrations [62]. This means that when the organism has more difficulty breaking the diet down and absorbing nutrients, the digestibility decreases. As a result, less energy is extracted from the diet, making less energy available to the animal.

Freire et al. 2000 [54], when analyzing the effects of diets supplemented with high levels of insoluble fiber fed to weaned piglets, such as soybean hulls or alfalfa meal, obtained significantly lower metabolizable energy values (12.95 and 13.24 MJ/kg, respectively) compared to a diet supplemented with beet pulp (14.23 MJ/kg), which has high nutrient digestibility. In addition, diets containing 15.6% or 29.7% wheat bran [68] and diets with 2% raw oat hulls [69] had lower digestible energy (4 to 8%) compared to the control diet.

Joven et al. [45], when replacing barley with increasing amounts of OC (0, 50, 100, and 150 g/kg of feed) in the diet of Duroc × (Landrace × Large White) pigs, observed a quadratic trend in which increasing levels of OC in the diet resulted in a reduction of DM and CF fractions in the ATTD, similar to the results found in our work. This reinforces the idea that most of these by-products are fibrous despite being rich in energy and nutrients. Adding these ingredients to pig diets modifies the composition of carbohydrates, reducing the starch content and increasing the presence of non-starch polysaccharides, the main components of dietary fiber. These results highlight the importance of paying attention to energy concentrations in diets with fiber-rich ingredients.

Current legislation in various countries restricts antimicrobial growth promoters and increases pressure to reduce antibiotic usage in animal production. In addition, understanding the relationship between immune status and nutritional requirements becomes essential to optimize animal performance. Therefore, adjusting feed formulations to optimize animal robustness and productive performance will be an increasingly relevant strategy. In this context, nutritionists should consider including components that strengthen the immune system when formulating diets for pigs. Although nutritional approaches can support animals’ resilience to health challenges and aid in disease recovery, avoiding strategies that induce excessive immune activation is equally essential, as this may compromise productive efficiency [70].

Various genetic and non-genetic factors influence the hematological parameters of livestock. Studies indicate that physiological, environmental, and nutritional aspects and fasting can alter the animals’ blood profile. Age, sex, drug administration, vitamin supplementation, and specific treatments also play a significant role. Other elements, including breed, climate, geographical location, season, level of physical activity, and health status, are equally determinant [71]. In newborn pigs, neutrophils account for approximately 60% to 85% of leukocytes, while lymphocytes represent around 20%. However, by about two weeks of age, an inversion occurs, with an increase in the proportion of lymphocytes and a reduction in neutrophils. This pattern persists throughout the animal’s life [72,73].

In this study, the hematological analysis of pigs fed diets containing different levels of OC revealed some statistically significant differences (*p* < 0.05) in specific parameters, particularly in lymphocytes, whose values increased in response to higher OC inclusion. This increase may be related to a moderate inflammatory response or an immune stimulus, although all values remained within the reference ranges reported in the literature [71,74,75,76]. It is essential to highlight that, to date, no specific hematological reference values have been established for the Bísaro breed, making further research necessary to characterize these parameters better. Consequently, the interpretation of the results followed the same criteria applied to domestic pigs.

Regarding the lipid profile, no significant variations were observed in total cholesterol, HDL, LDL, and triglyceride levels between treatments. However, an increasing trend in HDL levels (*p* = 0.055) was identified with including OC in the diet, which may indicate a beneficial effect on lipid metabolism.

The literature highlights that erythrocytes are highly susceptible to oxidative stress due to their role in oxygen transport. The development of oxidative stress can compromise hemoglobin integrity and lead to hemolysis, increasing free hemoglobin levels in plasma [77]. In this study, hemoglobin values and other hematological indicators showed no signs of oxidative damage, suggesting that OC inclusion in the diet did not induce significant oxidative stress. Although previous studies have reported a slight hemolytic effect in diets more enriched with agro-industrial by-products, our results do not indicate that the integrity of the erythrocytes is compromised, as the parameters analyzed remained within physiological standards.

To support this statement, it is essential to consider research investigating the effects of agro-industrial by-products on the hematological health of animals. For example, a study evaluating the use of cottonseed and soybean by-products in feedlot diets observed that including them did not negatively affect the animals’ blood parameters, indicating the feasibility of their use without compromising erythrocyte integrity [78].

Additionally, a review on agro-industrial by-products in animal nutrition highlights that crop residues such as cotton, sugarcane, peanuts, soybeans, and palm are potentially helpful as animal feed. Research on using these products as animal feed is necessary to meet the demand for animal nutrition and, indirectly, the dietary needs of a rapidly growing human population [79].

Thus, the findings of this study suggest that including OC in the diet of Bísaro pigs may be a viable and safe alternative, with no negative impacts on animal health. Furthermore, the potential positive influence on HDL levels suggests additional benefits for lipid metabolism. However, further studies are needed to assess long-term effects and validate using OCs as a sustainable ingredient in pig nutrition.

High-fiber diets significantly impact the intestinal villi of pigs, influencing their morphology and overall gut health. While these changes can positively contribute to increased nutrient absorption, the specific effects may vary depending on the type of fiber included in the diet. Diets with a high fiber content often promote an increase in villus height and the villus–crypt ratio in the intestines of pigs, indicating improved nutrient absorption and gut health. For instance, studies with diets containing high levels of insoluble fibers, such as corn bran and dried distillers’ grains, have shown an increase in villus height in the duodenum and ileum [59,80]. Similarly, flaxseed meal and oat hulls contribute to greater villus height in the jejunum and an improved villus–crypt ratio [81]. In our results, we observed no significant differences, but villi height and the villus–crypt ratio presented higher values in the OC15 group compared to the other two treatments. This may represent a positive effect of incorporating moderate quantities of OC.

The main components of dietary fiber, such as cellulose, hemicellulose, and pectin, constitute the primary elements of plant cell walls. These compounds can be fermented by various microorganisms present in the hindgut of mammals. Conversely, lignin, a high-molecular-weight polymer, is resistant to degradation in the digestive tract [59]. According to Hedemann, M.S. et al. [82], including pectin in pig diets resulted in shorter villi and crypts in the small intestine. However, the villus height–crypt depth ratio was unaffected. This effect may be related to the reduced feed intake observed with the pectin-containing diet, but it could also be due to potential direct impacts of this component. In contrast, diets rich in insoluble fibers improved intestinal morphology by increasing villus length, suggesting a beneficial effect on nutrient absorption. Furthermore, pigs fed these diets showed higher enzymatic activity in the mucosa and increased mucin content, indicating better protection against pathogenic bacteria than those fed diets rich in soluble fibers [82].

Despite the potential benefits of OC for gut health, there is a scarcity of studies in the literature explicitly evaluating the effects of its direct inclusion in the diets of Bísaro pigs. Most research focuses on other species or OC extracts, leaving a gap in the understanding of their impact on the gut microbiota, mucosal integrity, and digestive health parameters in this pig breed. This context highlights the need for further investigations to clarify the effects of OC on the intestinal health of Bísaro pigs, contributing to more sustainable and effective feeding practices.

## 5. Conclusions

Incorporating agro-industrial by-products, such as olive cake (OC), into animal feed presents a promising avenue for advancing sustainable livestock systems. By reducing environmental impacts, lowering feed costs, and minimizing competition for conventional resources, this strategy supports the principles of a circular economy, transforming waste into valuable nutritional inputs. In this study, the inclusion of 25% OC in the diet of Bísaro pigs was shown to impair nutrient digestibility (DM, OM, CP, CF, and NDF), leading to a reduced average daily gain (ADG) and an increased feed conversion ratio (FCR), despite a compensatory rise in feed intake. These findings highlight the importance of optimizing inclusion levels and processing methods when incorporating OC into swine diets. Nevertheless, inclusion levels of up to 15% proved nutritionally viable, balancing sustainability goals and animal performance.

Previous research with this autochthonous breed further supports these results, demonstrating that moderate OC inclusion does not compromise growth or meat quality, reinforcing its potential as a sustainable feed ingredient. However, economic feasibility should be evaluated, considering the cost of the OC and transportation, especially for compound feed factories, where the OC must be dried before use.

## Figures and Tables

**Table 1:** Ingredients and chemical composition of the experimental diets.

		Diets	
	OC0	OC15	OC25
Ingredients (g/100 g as fed basis)			
Olive cake	0	15	25
Barley	48.8	41.5	36.6
Wheat	18.1	15.4	13.6
Soybean meal 47	13.5	11.5	10.1
Rice bran	4.50	3.83	3.38
Corn germ	2.64	2.24	1.98
Corn grain	2.50	2.13	1.88
DDG’s corn	3.99	3.39	2.99
Beet molasses	3.50	2.98	2.63
Calcium carbonate	1.23	1.04	0.921
Monocalcium phosphate	0.116	0.099	0.087
Salt	0.283	0.240	0.212
Methionine	0.062	0.053	0.047
L-Lysine HCL	0.227	0.193	0.170
L-Threonine	0.080	0.068	0.060
Supplement min + vit + phytase	0.540	0.459	0.405
Chemical composition (% dry matter)			
Dry matter	88.8	90.3	91.3
Organic matter	93.6	94.2	94.6
Neutral detergent fiber	19.4	27.8	33.5
Acid detergent fiber	6.9	14.0	18.8
Acid detergent lignin	1.25	4.94	7.41
Crude protein	14.9	13.6	12.7
Crude fat	5.6	6.1	6.4
Phosphorus and Phytic Acid content (g/100 g)			
Total phosphorus	0.359	0.337	0.240
Phytic acid	1.27	1.20	0.85

OC0—control diet; OC15—control diet + 15% olive cake; OC25—control diet + 25% olive cake.

**Table 2:** Fatty acid profile of the experimental diets (g/100 g).

Fatty Acid Profile		Diets	
	OC0	OC15	OC25
C13:0	0.005	0.004	0.005
C14:0	0.753	0.701	0.407
C16:0	12.2	12.1	11.6
C18:0	3.08	3.02	2.68
C18:1n-9	24.1	26.4	38.9
C18:1n-7	1.50	1.55	1.83
C18:2n-6	31.0	29.8	23.3
C18:3n-3	1.73	1.69	1.43
C20:0	0.255	0.262	0.303
C20:1n-9	0.327	0.326	0.319
C22:1n-9	0.209	0.197	0.137
C24:0	0.213	0.214	0.217
ΣSFA	18.4	18.1	16.3
ΣMUFA	26.6	28.9	41.7
ΣPUFA	33.2	32.0	25.1
PUFA/SFA	1.80	1.76	1.54
n-6/n-3	16.0	15.7	14.1

OC0—control Diet; OC15—control Diet + 15% olive cake; OC25—control Diet + 25% olive cake; SFA—saturated fatty acid; MUFA—monounsaturated fatty acid; PUFA—polyunsaturated fatty acid; n-6/n-3 (∑ omega-6) (∑ omega-3).

**Table 3:** Granulometry analysis of the OC and diets.

Granulometry	Olive Cake	Diets		
	OC	OC0	OC15	OC25
Geometric mean diameter (μm)	645.7	205.1	250.4	294.4
Fineness modulus (mm)	2.63	0.98	1.27	1.50
Uniformity index (mm)				
>2	31.8	0.00	7.04	9.30
0.60 to 2	50.5	42.4	43.7	46.6
<0.60	17.7	57.6	49.2	44.1

Uniformity index values are determined in % of dry samples. OC—olive cake; OC0—control diet; OC15—control diet + 15% olive cake; OC25—control diet + 25% olive cake. >2—large particles; 2 to 0.60—medium particles; <0.60—small particles.

**Table 4:** Performances of Bísaro pigs fed different levels of OC.

		Diets		SEM	*p*-Value		
	OC0	OC15	OC25		ANOVA	Linear	Quadratic
iLW (kg)	91.4	92.1	91.4	3.29	0.984	0.988	0.862
fLW (kg)	122.4	128.9	121.6	2.19	0.056	0.987	0.018
ADG (g)	0.526	0.629	0.515	0.07	0.488	0.978	0.236
ADFI (g)	2.84 ^b^	3.46 ^a^	3.64 ^a^	0.19	0.036	0.013	0.566
FCR	4.64	5.01	6.59	0.87	0.295	0.168	0.476

iLW—initial live weight; fLW—final live weight; ADG—average daily gain; ADFI—average daily feed intake; FCR—feed conversion ratio; OC0—control diet; OC15—control Diet + 15% olive cake; OC25—control diet + 25% olive cake; SEM—standard error of the mean; different lowercase letters (a and b) in the same row correspond to significant differences between diets (*p* < 0.05). ANOVA followed by a post hoc Tukey test.

**Table 5:** Intake, apparent total tract digestibility, and digestible intake in Bísaro pigs fed different levels of OC.

	OC0	OC15	OC25	SEM	*p*-Value		
					ANOVA	Linear	Quadratic
Intake (g/Day)							
DM	2679.6	3402.6	3510.8	188.0	0.144	0.073	0.410
OM	2558.8	3217.8	3341.2	177.4	0.153	0.076	0.448
CP	392.6	502.6	467.3	26.2	0.230	0.246	0.197
CF	90.8 ^a^	171.5 ^b^	169.2 ^b^	13.1	0.002	0.002	0.025
NDF	586.8 ^a^	896.6 ^b^	1115.4 ^b^	75.2	0.002	0.001	0.614
Apparent Total Tract Digestibility (%)							
DM	83.7 ^b^	78.2 ^ab^	71.9 ^a^	1.71	0.003	0.001	0.838
OM	85.8 ^b^	79.5 ^ab^	72.8 ^a^	1.84	0.002	<0.001	0.929
CP	85.5 ^b^	84.5 ^ab^	79.3 ^a^	1.12	0.030	0.014	0.260
CF	91.2 ^b^	89.5 ^b^	82.6 ^a^	1.29	0.002	0.001	0.128
NDF	61.4 ^b^	46.9 ^ab^	37.4 ^a^	3.83	0.016	0.005	0.668
Digestible Intake (g/Day)							
DM	2246.9	2670.2	2530.6	147.5	0.536	0.466	0.406
OM	2199.0	2564.6	2436.6	140.8	0.606	0.527	0.450
CP	336.0	425.2	370.9	22.8	0.298	0.534	0.159
CF	82.5 ^a^	153.8 ^b^	140.0 ^b^	11.2	0.005	0.007	0.016
NDF	363.9	428.6	421.8	38.3	0.785	0.580	0.692

DM—dry matter; OM—organic matter; CP—crude protein; CF—crude fat; NDF—neutral detergent fiber. OC0—control diet; OC15—control diet + 15% olive cake; OC25—control diet + 25% olive cake; SEM—standard error of the mean; different lowercase letters (a and b) in the same row correspond to significant differences between diets (*p* < 0.05). ANOVA followed by a post hoc Tukey test.

**Table 6:** Blood parameters of Bísaro pigs fed different levels of OC.

	Diets			SEM	*p*-Value		
	OC0	OC15	OC25		ANOVA	Linear	Quadratic
Erythrogram							
Erythrocytes (10⁶/µL)	7.43 ^b^	8.05 ^ab^	8.65 ^a^	3.93	0.023	0.007	0.759
Hematocrit (%)	47.4	49.8	52.5	1.25	0.236	0.097	0.804
Microhematocrit (%)	44.0	45.4	45.6	0.02	0.721	0.439	0.845
Hemoglobin (g/dL)	14.7	15.3	16.2	0.35	0.559	0.649	0.333
MCV (fL)	63.7	61.9	61.1	0.75	0.358	0.160	0.874
MCH (pg)	19.8	19.1	18.8	0.21	0.162	0.062	0.771
MCHC (g/dL)	31.2	30.9	30.9	0.15	0.596	0.345	0.729
RDW (%)	22.0	23.1	23.2	0.32	0.274	0.124	0.676
Reticulocyte (%)	0.471	0.567	0.525	0.08	0.901	0.767	0.733
Reticulocyte Count (10^3^/µL)	36.0	48.5	45.6	6.83	0.766	0.557	0.673
Leucogram							
Leukocytes (10^3^/µL)	14.2	15.6	15.2	0.60	0.603	0.438	0.530
Neutrophil (%)	34.9	30.8	27.4	1.39	0.070	0.023	0.883
Lymphocyte (%)	51.3 ^b^	58.9 ^ab^	60.9 ^a^	1.51	0.011	0.004	0.519
Monocyte (%)	9.46 ^a^	7.07 ^b^	8.81 ^ab^	0.38	0.033	0.326	0.015
Eosinophil (%)	4.33	3.10	2.76	0.32	0.091	0.034	0.661
Basophil (%)	0.057	0.133	0.063	0.002	0.125	0.767	0.046
Neutrophil (10^3^/µL)	4.96	4.88	3.86	0.28	0.195	0.119	0.355
Lymphocyte (10^3^/µL)	7.16 ^b^	9.14 ^a^	8.48 ^a^	0.27	0.006	0.013	0.025
Monocyte (10^3^/µL)	1.36	1.11	1.21	0.06	0.294	0.282	0.253
Eosinophil (10^3^/µL)	0.626	0.483	0.384	0.05	0.110	0.038	0.978
Basophil (10^3^/µL)	0.007	0.018	0.006	0.002	0.100	0.999	0.034
Pratelets and Coagulation							
Platelet (10^3^/µL)	174.6 ^a^	98.6 ^b^	119.5 ^ab^	2.00	0.024	0.025	0.086
MPV (fL)	13.3	13.6	12.7	0.21	0.232	0.307	0.168
Plateletcrit (%)	0.225 ^a^	0.108 ^b^	0.150 ^ab^	0.48	0.020	0.042	0.038
PT (seconds)	12.7	13.8	14.8	0.13	0.447	0.212	0.959
INR	1.03	1.16	1.27	0.70	0.445	0.212	0.930
APTT (seconds)	13.1	15.8	14.8	0.08	0.204	0.165	0.255
Lipid profile							
Total Cholesterol (mg/dL)	106.0	112.6	118.4	3.05	0.274	0.113	0.900
HDL (mg/dL)	46.9	44.1	47.9	1.02	0.299	0.779	0.131
LDL (mg/dL)	54.3	52.9	55.8	1.08	0.559	0.649	0.333
Triglycerides (mg/dL)	56.7	58.5	65.0	5.11	0.806	0.560	0.777
Biochemical Parameters							
ALT (U/L)	48.7	46.1	46.0	0.62	0.900	0.680	0.851
GGT (U/L)	39.8	44.5	35.6	2.50	0.422	0.627	0.227
AST (U/L)	33.4	44.1	32.0	2.81	0.692	0.985	0.398
Alkaline Phosphatase (U/L)	101.2	105.4	94.4	6.27	0.729	0.689	0.498
Total Bilirubin (mg/dL)	0.020	0.027	0.031	5.62	0.305	0.129	0.919
Protein (g/dL)	6.66	6.50	6.75	0.88	0.770	0.818	0.499

MCV—mean corpuscular volume; MCH—mean corpuscular hemoglobin; MCHC—mean corpuscular hemoglobin concentration; RDW—red cell distribution width; MPV—mean platelet volume; PT—prothrombin time; INR—international normalized ratio; APTT—activated partial thromboplastin time; HDL—high-density lipoprotein; LDL—low-density lipoprotein; ALT—alanine aminotransferase; GGT—gamma-glutamyl transferase; AST—aspartate aminotransferase; OC0—control diet; OC15—control diet + 15% olive cake; OC25—control diet + 25% olive cake; SEM—standard error of the mean; different lowercase letters (a and b) in the same row correspond to significant differences between diets (*p* < 0.05). ANOVA followed by a post hoc Tukey test.

**Table 7:** Villi development in the ileum of Bísaro pigs fed different levels of OC.

Measurements	Diets			SEM	*p*-Value		
	OC0	OC15	OC25		ANOVA	Linear	Quadratic
Ileum							
Villi Height (µm)	328.1	362.3	348.2	11.15	0.355	0.518	0.201
Villi Width (µm)	120.8	134.7	123.9	3.43	0.506	0.274	0.711
Crypt Depth (µm)	353.1	313.3	332.0	11.68	0.483	0.667	0.265
Villus–Crypt Ratio (µm)	1.04	1.22	1.08	0.06	0.394	0.535	0.227
Absorption (mm^2^)	0.13	0.15	0.14	0.01	0.211	0.417	0.117
Jejunum							
Villi Height (µm)	380.0	398.8	360.6	11.62	0.698	0.657	0.475
Villi Width (µm)	118.0	120.3	123.8	2.29	0.280	0.543	0.143
Crypt Depth (µm)	345.2	305.6	328.5	11	0.212	0.743	0.087
Villus–Crypt Ratio (µm)	1.14	1.41	1.15	0.06	0.465	0.255	0.644
Absorption (mm^2^)	0.14	0.15	0.14	0.01	0.963	0.866	0.831

OC0—control diet; OC15—control diet + 15% olive cake; OC25—control Diet + 25% olive cake; SEM—standard error of the mean.

## Data Availability

Data are contained within the article.

## Abbreviations

The following abbreviations are used in this manuscript:

OC	Olive Cake
ATTD	Apparent Total Tract Digestibility
DM	Dry Matter
OM	Organic Matter
CP	Crude Protein
CF	Crude Fat
NDF	Neutral Detergent Fiber
ADF	Acid Detergent Fiber
ADL	Acid Detergent Lignin
SFA	Saturated Fatty Acid
MUFA	Monounsaturated Fatty Acid
PUFA	Polyunsaturated Fatty Acid
AST	Aspartate Aminotransferase
ALT	Alanine Aminotransferase
GGT	Gamma-Glutamyl Transferase
ALP	Alkaline Phosphatase
HE	Hematoxylin and Eosin
VH	Villus Height
CD	Crypt Depth
iLW	Initial Live Weight
fLW	Final Live Weight
ADG	Average Daily Gain
ADFI	Average Daily Feed Intake
FCR	Feed Conversion Ratio
SEM	Standard Error of the Mean
MCV	Mean Corpuscular Volume
MCH	Mean Corpuscular Hemoglobin
MCHC	Mean Corpuscular Hemoglobin Concentration
RDW	Red Cell Distribution Width
MPV	Mean Platelet Volume
PT	Prothrombin Time
INR	International Normalized Ratio
APTT	Activated Partial Thromboplastin Time
HDL	High-Density Lipoprotein
LDL	Low-Density Lipoprotein
